# A Wrapper Feature Subset Selection Method Based on Randomized Search and Multilayer Structure

**DOI:** 10.1155/2019/9864213

**Published:** 2019-11-04

**Authors:** Yifei Mao, Yuansheng Yang

**Affiliations:** School of Computer Science and Technology, Dalian University of Technology, Dalian, China

## Abstract

The identification of discriminative features from information-rich data with the goal of clinical diagnosis is crucial in the field of biomedical science. In this context, many machine-learning techniques have been widely applied and achieved remarkable results. However, disease, especially cancer, is often caused by a group of features with complex interactions. Unlike traditional feature selection methods, which only focused on finding single discriminative features, a multilayer feature subset selection method (MLFSSM), which employs randomized search and multilayer structure to select a discriminative subset, is proposed herein. In each level of this method, many feature subsets are generated to assure the diversity of the combinations, and the weights of features are evaluated on the performances of the subsets. The weight of a feature would increase if the feature is selected into more subsets with better performances compared with other features on the current layer. In this manner, the values of feature weights are revised layer-by-layer; the precision of feature weights is constantly improved; and better subsets are repeatedly constructed by the features with higher weights. Finally, the topmost feature subset of the last layer is returned. The experimental results based on five public gene datasets showed that the subsets selected by MLFSSM were more discriminative than the results by traditional feature methods including LVW (a feature subset method used the Las Vegas method for randomized search strategy), GAANN (a feature subset selection method based genetic algorithm (GA)), and support vector machine recursive feature elimination (SVM-RFE). Furthermore, MLFSSM showed higher classification performance than some state-of-the-art methods which selected feature pairs or groups, including top scoring pair (TSP), k-top scoring pairs (K-TSP), and relative simplicity-based direct classifier (RS-DC).

## 1. Introduction

Identifying disease types/subtypes from biomedical data is very important to understand diseases and develop drugs, among other important functions. In this context, many machine-learning techniques, including support vector machine (SVM) [1], random forest (RF) [2], and *k*-nearest-neighbor (KNN) [3], have been applied in this field with remarkable performance [4, 5].

Given that biomedical data are expensive to generate and difficult to obtain, a small number of samples with thousands of features would distort the distribution of the real data. Various selection methods had been proposed to remove the insignificant features and improve the prediction performance of the models [6–9]. Depending on the way to combine the search of feature subsets with the construction of classification model, feature selection methods are divided into three categories: filter methods, wrapper methods, and embedded methods [10]. Filter methods focus on univariate or multivariate analysis and ignore the interaction with classifier. *T*-test, relief [11], correlation-based feature selection (CFS) [12], and fast correlation-based feature selection (FCBF) [13] are the common filter methods. Different from filter methods, wrapper methods use classification models to evaluate the selected feature subsets, including sequential search [14], genetic algorithms (GA) [15], and estimation of distribution algorithm (EDA) [16]. Embedded methods select optimal feature subsets and construct suitable classification models simultaneously. Support vector machine recursive feature elimination (SVM-RFE) is a typically famous embedded feature selection method [17].

As the number of feature subsets would increase exponentially with the number of features, it is impractical to evaluate all the subsets in wrapper or embedded methods. Search strategies had been proposed. Among these strategies, randomized and deterministic methods were the most frequently used [18–20]. Randomized methods search the subsets with some kind of randomness, including the Las Vegas wrapper (LVW) [21], genetic algorithm partial least squares (GAPLS) [22], and Monte Carlo-based uninformative variable elimination in partial least squares [23], while deterministic methods do with some heuristic search ways, including sequential forward selection (SFS) and sequential backward selection (SBS).

However, deterministic methods were often prone to local optimum while randomized search method always returned the ranking of features. Because the upper limit on the number of features in a subset was difficult to be predetermined, the complexity of randomized search methods would increase exponentially with the upper limit. In this paper, we propose a wrapper feature selection method with randomized search strategy. Unlike other randomized search methods, the goal of our method is to select a feature subset. We employ randomized search and multilayer structure to constantly adjust the weights of each feature. First, all the features are assigned same weights. Many feature subsets are generated based on the weights, and the classification models are constructed with SVM for each subset. The weight of a feature should increase if it is selected into more subsets with better performance than other features on the current layer. In this manner, the weights of features are revised layer-by-layer, precision of feature weights are constantly improved, and better subsets are repeatedly constructed by the features with higher weights. Finally, the topmost feature subset of the last layer is returned as the result. Herein, our multilevel feature subset selection method (MLFSSM) is compared with LVW, GAANN [24], SVM-RFE, and other feature selection methods using publicly available cancer datasets.

## 2. Methods

We assumed a dataset *X* (*N* × *M*), where *N* is the number of samples and *M* is the number of features. The feature set is denoted as *F* = {*f*_1_, *f*_2_,…, *f*_*M*_}, and the class label set *C* is denoted as *C* = {−1,1}.

In this article, we propose a multilayer feature subset selection method named MLFSSM. First, the features are set to the same weight and picked into the subsets based on the weights. To obtain diverse feature combinations, many subsets are generated to assure the diversity of the combinations. Subsequently, classification models are constructed on each subset. According to the accuracies of the models, the weight of a feature should be increased if it is selected into more subsets with better performance than other features on the current layer. In this way, the weights of features are recalculated, and new subsets are regenerated using the weights on the following layer. The process is repeated until the terminal condition is met. The subset with the highest classification accuracy among those on the last layer is returned as the final result. Here, (1) how to calculate feature weights, (2) how to select features into the subsets, and (3) how to decide the terminal condition are the three key issues.

(1) Considering the issue of calculation of feature weights, the features in the subset might be more discriminative than others when a feature subset achieves a high accuracy rate, and their weights should be increased for the next subset selection. Furthermore, the weights of features calculated on the former layers are involved in the computation of the feature weights on the current layer. The weight of feature *f* on layer *l* is calculated as follows:
  (1) $$\begin{array}{c} w_{l,f}=\left\{\begin{array}{cc} \frac{1}{M}, & \text{if }l=1,\\ \alpha *w_{\;l-1,f}+\overset{M}{\underset{m=1}{\sum }}{\left({\text{accu}}_{ft_{l,m}}*{\text{flag}}_{m}\right)}^{p}, & \text{if }l\;\geq \;2. \end{array}\right. \end{array}$$
For diversity feature subsets, the total number of subsets generated on layer *l* equals to the number of features *M*. *ft*_*l*,*m*_ denotes the *m*^th^ subset on the *l*^th^ layer, and accu_*ft*_*l*,*m*__ is the classification accuracy of *ft*_*l*,*m*_ on layer *l*. *w*_ *l*−1,*f*_ is the weight of feature *f* on the former layer *l* − 1. And

(2) $$\begin{array}{c} {\text{flag}}_{m}=\left\{\begin{array}{cc} 1, & \text{if }f\in ft_{l,m},\\ 0, & \text{else}. \end{array}\right. \end{array}$$

  Each subset *ft*_*l*,*m*_ (1 ≤ *m* ≤ *M*) includes *ls* features (the length of the subset). On the first layer, we set each subset contains nonduplicate features, and the occurrence frequency of each feature is equal. On the following layers, *M* subsets were constructed by the revised feature weights, while duplicate features might be contained. We will discuss how to decide the appropriate value of *ls* in the experimental section.
  Furthermore, apt values of *α* and *p* might prevent the program from getting stuck in a local optimum and learn enough information from the former layers at the same time. In the experimental section, we will discuss how to choose the appropriate value of the parameters in the experimental section.
(2) For the second issue, the probability of feature *f* being selected on layer *l* is

(3) $$\begin{array}{c} {\text{pos}}_{l,f}=\frac{W_{l,f}}{\sum_{j=1}^{M}w_{l,j}}. \end{array}$$

  From equation (3), it is observed that features have equal probabilities 1/*M* for selection on the first layer. With the weights being revised, the features with higher weights would appear in more subsets for their larger posibility values than other features.
(3) For the terminate condition issue, although more layers might achieve higher performance, the running time will sharply increase and the performance improvement will slow down or stabilize with the number of layers. The algorithm could be terminated when the accuracy rate of the top *T* feature subset reaches 100% or when the number of layers reaches *L.* Here, we suggest *T* = 20 and *L* = 20 for tolerable running time and enough stability results.

Algorithm 1 lists the description of the MLFSSM algorithm.

## 3. Experiments

To validate the effectiveness of MLFSSM, we attempt to discuss three issues:

1. In this method, three parameters affect the performance of MLFSSM, including weight ratio *α* and power number *p* in equation (1) and the length of every subset *ls*.
2. The performances of MLFSSM, LVW, GAANN, SVM-RFE, and other traditional feature selection methods are compared to assess whether MLFSSM is more effective than the other methods.
3. The performance of MLFSSM is compared with those of the three methods, including TSP, K-TSP, and RS-DC. We further validate whether the subset selected by MLFSSM is more effective than the pairs or groups by the other methods.

In this study, SVM is used for classification. The codes of SVM were downloaded from LibSVM (available at http://www.csie.ntu.edu.tw/∼cjlin/libsvm). RBF kernel was used for good bioinformatics performance, and the penalty parameter C was set to 1 in SVM-RFE and MLFSSM. All the experiments used 20 times five-fold cross validation. Five public gene datasets were used in the three experiments.  Table 1 lists the details of the datasets.

### 3.1. Effects of Parameters

The default values and the ranges of the three parameters are listed in Table 2. To study the effects of the parameters, the value of one parameter was changed at a time, while the values of other parameters were set to the default values in the experiments.

#### 3.1.1. Effects of Weight Ratio *α*

**Figure 1:**
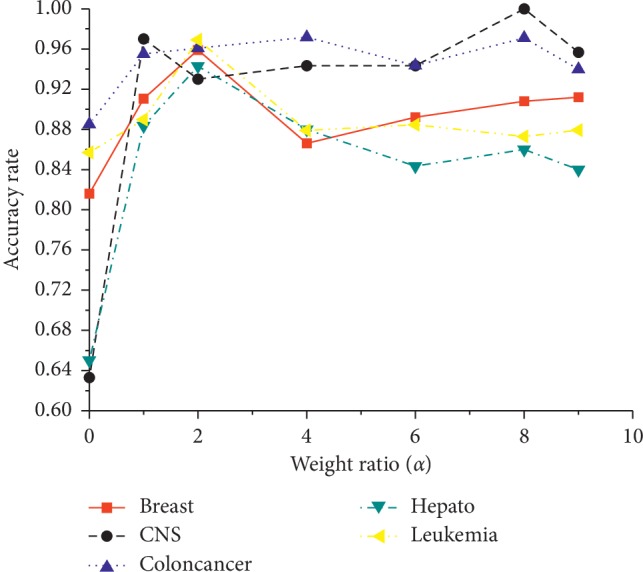
Effects of weight ratio *α*.

Figure 1 shows the effects of the ratios *α* in MLFSSM which ranges from 0.1 to 0.9. It is observed that the values are usually lower than others when *α* = 0.1 or 0.9, and the accuracies at *α* = 0.2 show well above the ones at other values in most of the datasets. The possible reason could be that *α* = 0.2 could get a globally optimal solution and avoid falling into local optimum in MLFSSM. So *α* = 0.2 is suggested as the default value.

#### 3.1.2. Effects of Power Number *p*

**Figure 2:**
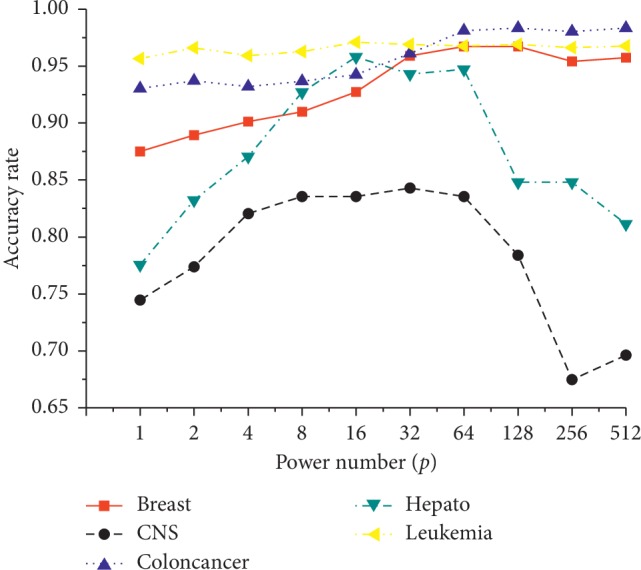
Effects of power number *p*.

Figure 2 shows the effects of power number *p*, which ranges from 1 to 512. The accuracy usually increases when *p* ≤ 32 with the highest accuracies at *p*=32. Subsequently, the accuracy often decreases when *p* > 32. This might be because smaller or larger *p* values fail to find the global optimum features. Thus, *p*=32 is suggested as the default value.

#### 3.1.3. Effects of Feature Subset Length *ls*

**Figure 3:**
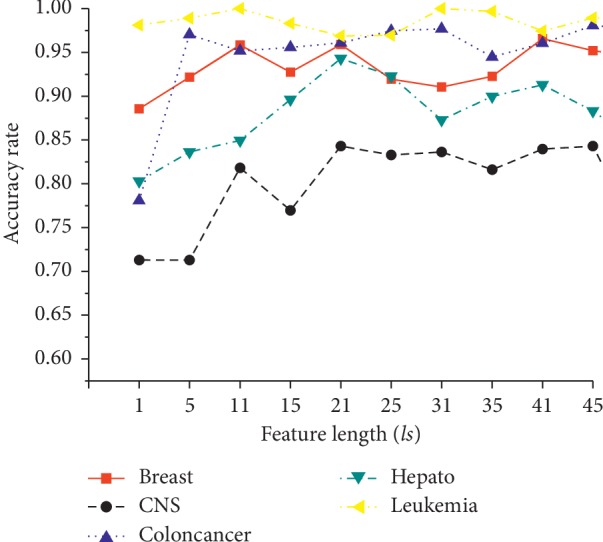
Effects of feature subset length *ls*.

Figure 3 shows the effects of feature subset length *ls*, which ranges from 1 to 51. The accuracies at *ls* = 21 have showed better performances than the ones at other values in all five datasets. The possible reason could be that a modest number of features not only include informative features but also exclude noise features. Therefore, *ls* = 21 is suggested as the default value.

### 3.2. Comparison with LVW and Its Improved Method

LVW is a typical wrapper feature selection method [21]. It was proposed by Liu and Setiono which used the Las Vegas method for randomized search strategy to select feature subsets. The description of LVW is listed in Supplementary [Supplementary-material supplementary-material-1]. For comparisons, we set *T* = *M* ∗ 21 as one termination condition of LVW.

Furthermore, we improved LVW with constantly revised weights in randomized search procedure of LVW named imp-LVW. In imp-LVW, feature weights were equal to each other firstly. Furthermore, the weight of a feature would increase if the current subset including the feature has better performance than the previous subsets. The description of imp-LVW is listed in Supplementary [Supplementary-material supplementary-material-1]. Similar to LVW, we set *T* = *M* ∗ 21 as one termination condition of imp-LVW.

**Figure 4:**
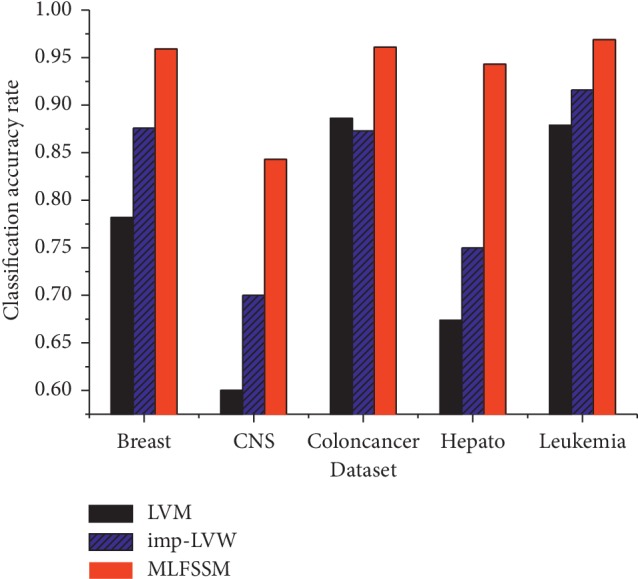
Comparisons in LVW, imp-LVW, and MLFSSM.

Figure 4 shows the classification accuracy rates of MLFSSM, LVW, and imp-LVW in the five public datasets. As three wrapper feature selection methods, LVW always has the lowest values in the methods, imp-LVW shows the better performance than LVW, and MLFSSM displays the best performance in the methods. The possible reason might be the constantly revised weights bring improvement in the performance. Because the weights of features stay consistent over time in LVW, the optimal subset is difficult to be found in a limited time for large dimensions of biomedical data. Imp-LVW changes the weights continuously. However, imp-LVW only focuses on the performance of the current subset which might fall into the local optimum. MLFSSM constantly adjusts the weights of each feature with the layers. A feature would be selected in the final subset based on good performance in not only the current layer but also the former layers.

### 3.3. Comparison with Fuzzy_GA and GAANN

In this section, we compare MLFSSM with Fuzzy_GA [30] and GAANN [24] based on genetic algorithm. Fuzzy_GA was proposed by Carlos et al. Fuzzy_GA combined fuzzy systems and genetic algorithm to classify Wisconsin breast cancer database (WBCD) dataset involving a few simple rules. GAANN was proposed by Fadzil et al. It used genetic algorithm (GA) for feature subset selection and parameter optimization of an artificial neural network (ANN). In addition, three variations of backpropagation were applied for GAANN and GAANN with backpropagation (GAANN_RP) showing the best accuracies.

The comparison uses WBCD dataset as Ahmad et al. did [24]. Moreover, we replaced the missing values, rescaled the attributes, and used cross-validation methods in the experiments as Ahmad et al. did [24].

Table 3 shows the average accuracies of Fuzzy_GA, GAANN_RP, and MLFSSM. We could observe that MLFSSM shows the best performance in the methods. Based on GA, Fuzzy_GA and GAANN_RP generate a new population (subset) by crossover and mutation using two chromosomes in each generation. There are two important factors in MLFSSM different from them: one is a large number of feature subsets generated in each layer; the other is the method of feature evaluation based on multilayer. The two factors not only guarantee informative feature subsets selected, but also avoid premature convergence and instability results.

### 3.4. Comparison with Traditional Feature Selection Methods

In this section, we describe the comparison of MLFSSM with some feature selection methods on the five public datasets. The comparative methods, including SVM-RFE, least square-bound (LS-Bound) [6], Bayes + KNN [7], elastic net-based logistic regression (EN-LR) [31], guided regularized random forest (GRRF) [32], and T-SS [33] had shown improved performance in biomedical data in recent years. The results of the comparison of the methods have been previously reported [33].  Table 4 shows the average accuracy rates of the methods. In the table, the bold and italic numbers indicate the largest values using the corresponding method in a dataset.

In Table 4, MLFSSM shows superiority over the four feature selection methods for the five datasets. We observe that the highest accuracy rates among these methods are 0.693 (by T-SS) and 0.693 (by SVM-RFE), respectively, for Hepato and CNS datasets, which is well below those by MLFSSM (by 0.25 and 0.15, respectively). As the compared methods are based on deterministic search strategies, the results show the effectiveness of MLFSSM with randomized search strategy.

### 3.5. Comparison with the Methods Selecting Pairs or Groups

In this section, we compare MLFSSM with TSP [34], K-TSP [35], and RS-DC [36] to discuss whether the subsets selected by MLFSSM are more effective than the pairs or groups selected by the other methods. TSP was proposed by Geman et al. This method focused on pairwise rank comparisons to reflect the underlying biological role and selected the top feature pair to build a classification model when the two features of the pair shifted their rank positions more dramatically in the phenotypic classes than others. Because one feature pair might not contain enough information, Tan et al. suggested selecting top *K* feature pairs for building *K* classification models and ensembling the final classification results by majority voting. The *K* value should not be too large; thus, it was often set from 3 to 11. Given that the length of subsets is suggested to be 21 in MLFSSM, we set *K* = 11 in K-TSP for comparison. Chen et al. integrated individual feature effects with pairwise joint effects between the target feature and others, proposed a novel score measure named relative simplicity (RS), and built RS-DC to select binary-discriminative genes for classification.  Table 5 lists the average accuracy rates of the four compared methods using the five datasets, and the bold and italic numbers indicate the largest values using the corresponding method in a dataset.

Table 5 shows that MLFSSM has obvious advantages over TSP, K-TSP, and RS-DC in Breast, Hepato, and CNS. MLFSSM showed outstanding performance in Hepato and CNS, where MLFSSM achieved 0.943 and 0.843, respectively, for accuracy, which are higher by 0.286 and 0.246 points than the maximum values by TSP, K-TSP, and RS-DC. The possible reason could be that these three methods only focused on the discriminative ability of pairs, while MLFSSM could find the informative feature subsets with more than two features.

### 3.6. Analysis of the Selected Top Feature Pairs

In this section, we further analyze the ten most selected genes of the final subsets in CNS.  Table 6 lists the details of the features and the corresponding biological pathway using [37].

**Figure 5:**
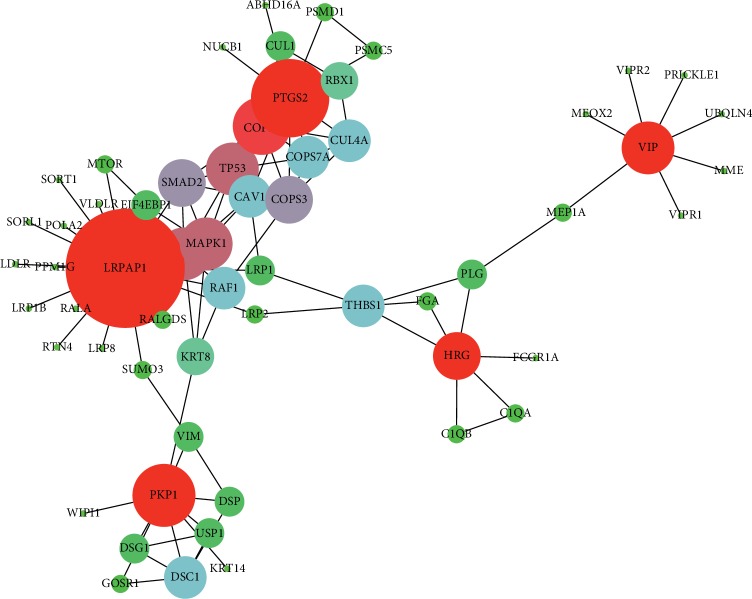
Gene interaction diagram. Note: the size of node is related with its degree in the graph.

Figure 5 shows the interaction of the genes (red nodes) selected by us with the other nodes (other color nodes) identified by researchers from outside the selected dataset. We could find that the identified genes are all top 5-ranked ones in Table 6, and they have minimum 4 interactions. Especially, LRPAP1 shows the highest degree in Figure 5, which have been proved as a valuable marker in many diseases such as gallbladder cancer [38], Alzheimer disease [39], and lymphoma [40].

## 4. Discussion

In this paper, we focus on searching discriminative feature subsets. For this to be realized, it is crucial to have a large amount and diversity of subsets. At the first layer, we initialize features with equal weights of appearance and construct subsets whose quantity is the same as feature number. Meanwhile, the length of the subsets (*ls*) is long enough to provide diverse feature combinations and appropriate runtime. In the experimental section, we show *ls* = 21 brings better performance than other values.

**Figure 6:**
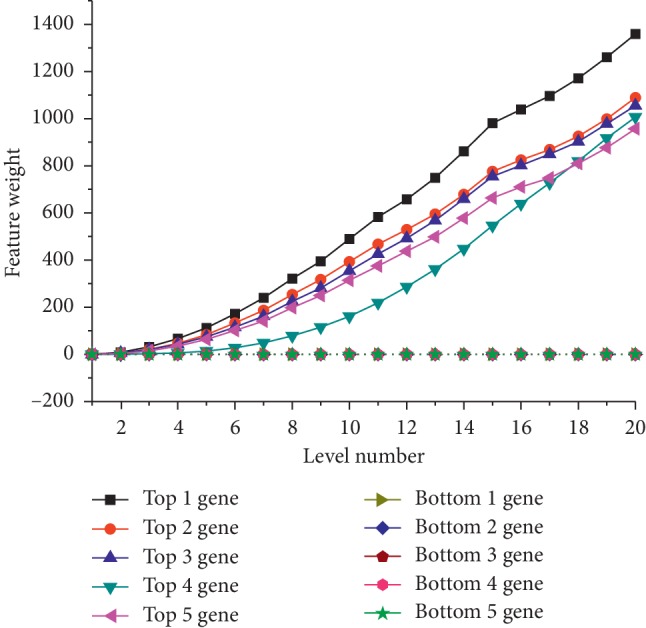
Weight values with increasing layer of top and bottom 5-ranked genes on CNS.

**Figure 7:**
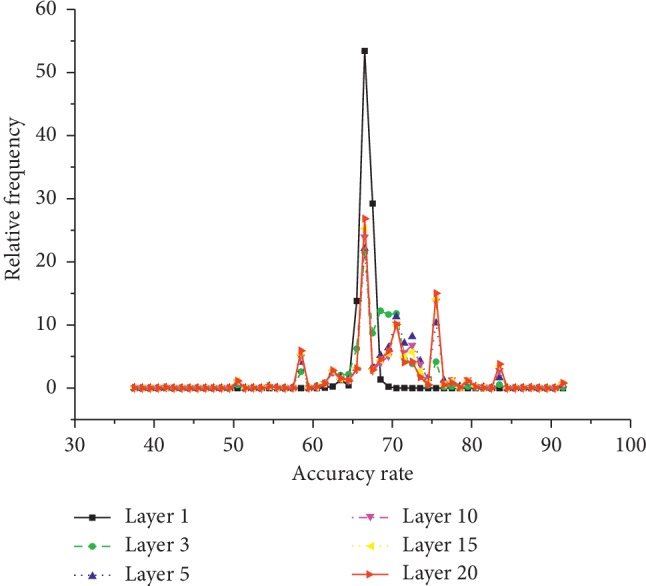
The frequencies of accuracy rates of features with increasing layer on CNS.

Based on the multilayer structure, we revise feature weights and find good subset gradually. Next, we will take CNS dataset as an example to further show the influence of multilayer structure on feature subset selection. MLFSSM shows the highest accuracy rate on CNS dataset among the comparative methods. We could find the average total level on CNS dataset as 18.29, which is far above the ones on other datasets. The possible reason is that MLFSSM evaluates feature weights with amounts of subsets in each layer, revises the values of features by their performances on former and current layers, and selects the highest performances of feature combinations through multilayer structure. Next, we will further analyze the classification procedures of MLFSSM on the CNS dataset with Figures 6 and 7.

Figure 6 shows the influence on the weights of 10 features in CNS dataset including top-ranked 5 and bottom-ranked 5 features at the last layer. It is observed that the weights of the features are equal at layer one. The top 5-ranked features are continually revised to higher weights and the bottom 5-ranked features are revised to lower weights with the increase of layer number.

Then, we further analyze the accuracy rates of features on different layers in Figure 7. If feature *f*  ∈  *ft*_*l*,*m*_, the accuracy rate of subset *ft*_*l*,*m*_ is averaged as the accuracy rate of feature *f* on layer *l*. We make a statistical analysis on the frequency of different accuracy rates with increasing layers. We could observe that the accuracy rates of 99% features are about 60% in layer 1, and then the rates of 28.37% features increase to over 70% in layer 2. With increasing layer, the rates of some features increase. Finally, the rates of 0.78% features are over 90% in the last layer. The result further shows that MLSFFM with a multilayer structure obtains more accurate feature evaluations and more effective feature subsets.

## 5. Conclusion

Here, we propose a wrapper feature subset method called MLFSSM, wherein based on the multilayer structure, we compute the weights of features and generate subsets by weights layer-by-layer. Ultimately, the top feature subset of the last layer is returned. Experiments on five public gene datasets showed MLFSSM to have an advantage over other similar methods in terms of classification performance. In the future, we plan to further analyze the features for biomarker detection, ascertain how to dynamically determine the parameter values on different datasets, and improve the running speed of the algorithm.

## Figures and Tables

**Algorithm 1:**
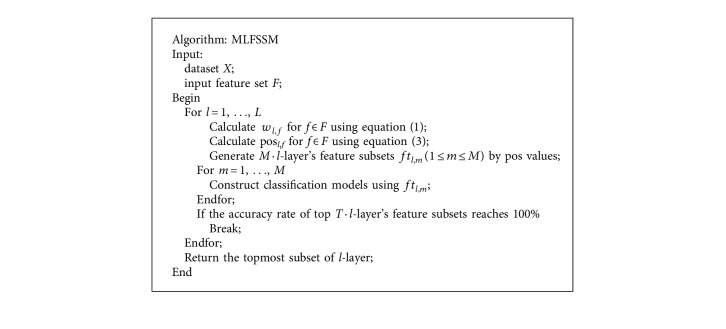
Description of the MLFSSM algorithm.

**Table 1:** Details of five public datasets for comparison.

No.	Dataset	Feature number	Sample number
1	Breast [25]	7129	49
2	ColonCancer [26]	2000	60
3	CNS [27]	7129	60
4	Hepato [28]	7129	60
5	Leukemia [29]	7129	72

**Table 2:** Summary of the parameter setting.

Parameters	Default values	Range
Weight ratio *α*	0.2	0.1, 0.2, 0.4, 0.6, 0.8, 0.9
Power number *p*	32	1, 2, 4, 8, 16, 32, 64, 128, 256, 512
Feature subset length *ls*	21	1, 5, 11, 15, 21, 25, 31, 35, 41, 45, 51

**Table 3:** Comparison of the accuracies of GAANN_RP and MLFSSM.

Method	Average accuracy
Fuzzy_GA	0.9736
GAANN_RP	0.9829
MLFSSM	**0.9997**

**Table 4:** Comparison of the average accuracy rates of MLFSSM with four feature selection methods.

Method	Breast	Leukemia	Colon	Hepato	CNS
SVM-RFE	0.877	0.967	0.835	0.658	0.693
LS-bound	0.778	0.935	0.817	0.618	0.61
Bayes + KNN	0.821	0.92	0.828	0.628	0.628
EN-LR	0.854	0.962	0.837	0.683	0.664
GRRF	0.846	0.939	0.834	0.67	0.618
T-SS	0.893	**0.97**	0.871	0.693	0.655
MLFSSM	**0.959**	0.969	**0.961**	**0.943**	**0.843**

**Table 5:** Comparison of the average accuracy rates of MLFSSM with four feature selection methods based on groups.

Method	Breast	Leukemia	Colon	Hepato	CNS
TSP	0.783	0.900	0.891	0.602	0.496
K-TSP (*K* = 11)	0.870	**0.969**	**0.962**	0.657	0.517
RS-DC	0.868	0.944	0.896	0.604	0.597
MLFSSM	**0.959**	**0.969**	0.961	**0.943**	**0.843**

**Table 6:** Details of the ten most selected genes of CNS dataset.

No.	Gene accession number	Gene description	Official symbol	Gene ID	Biological pathway
1	M13149_at	HRG histidine-rich glycoprotein	HRG	3273	Dissolution of fibrin clot
2	S75989_at	Gamma-aminobutyric acid transporter type 3 (human, fetal brain, mRNA, 1991 nt)	—	—	—
3	HG2987-HT3136_s_at	Vasoactive intestinal peptide	VIP	7432	Glucagon-type ligand receptors
4	M63959_at	LRPAP1 low density lipoprotein-related protein-associated protein 1 (alpha-2-macroglobulin receptor-associated protein 1)	LRPAP1	4043	Reelin signaling pathway Lissencephaly gene (LIS1) in neuronal migration and development
5	Z73677_at	Gene encoding plakophilin 1b	PKP1	5317	Apoptotic cleavage of cell adhesion proteins Apoptotic cleavage of cellular proteins
6	D79986_at	KIAA0164 gene	—	—	—
7	M23575_f_at	PSG11 pregnancy-specific beta-1 glycoprotein 11	PSG11	5680	—
8	U37139_at	Beta 3-endonexin mRNA, long form and short form	—	—	—
9	D28235_s_at	Cyclooxygenase-2 (hCox-2) gene	PTGS2	5743	COX reactions Prostanoid metabolism Calcium signaling in the CD4+ TCR pathway JNK signaling in the CD4+ TCR pathway Ras signaling in the CD4+ TCR pathway
10	HG2271-HT2367_at	Profilaggrin	—	—	—

## Data Availability

Previously reported public datasets (including Breast, ColonCancer, CNS, Hepato, and Leukemia) are included within the supplementary information files.

## Abbreviations

GA: Genetic algorithm
SVM-RFE: Support vector machine recursive feature elimination
MLFSSM: Multilevel wrapper feature subset selection method
RF: Random forest
KNN: *k*-nearest-neighbor
LVW: Las Vegas wrapper
LS-Bound: Least square-bound
EN-LR: Elastic net-based logistic regression
GRRF: Guided regularized random forest
TSP: Top scoring pair
K-Tsp: k-top scoring pairs
TST: Top scoring triplet
TSN: Top scoring *n*
RS-DC: Relative simplicity-based direct classifier.

## Supplementary Materials

Supplementary Materials Public datasets: details of five public datasets (had been described in Table 3 in the paper) which are the important datasets for classification with our methods and other comparative methods. Supplymentary Tables 1 and 2: description of two algorithms (LVW and Imp-LVW) which were compared with our method in Section 3.2.
