# Supplementary material for: A Wrapper Feature Subset Selection Method Based on Randomized Search and Multilayer Structure
Source: Biomed Res Int. 2019 Nov 4;2019:9864213. doi: 10.1155/2019/9864213 (PMC6885241; doi:10.1155/2019/9864213)
Supplement: Supplementary Materials — Public datasets: details of five public datasets (had been described in Table 3 in the paper) which are the important datasets for classification with our methods and other comparative methods. Supplymentary Tables 1 and 2: description of two algorithms (LVW and Imp-LVW) which were compared with our method in Section 3.2. [file 9864213.f1.zip › 9864213.f1/20190426-supplymentary table1-2.docx]

TABLE 1
Description of LVW

| Algorithm: LVW  Input:  dataset *X*;  input feature set *F*;  maximum loop number $t_{max};$  Begin  $E=\infty$*;*  $F^{*}=F$*;*  *d*=21;  *t=0;*  $T=\left\vert F \right\vert\times21$*;*  Initialize feature weight *w*, for each$f\in F$*,* $w_{f}=1$(where \|*w*\|=\|*F*\|);  For *loopnum*=1: *T*  generate feature subset $F^{'}$ with 21 features by feature weights $w$ using randomize method;  delete duplicate features from $F^{'}$  $d^{'}=\left\vert F^{'} \right\vert;$  *E’* is the classification accuracy of $F^{'}$on SVM model;  If *(*$E^{'}<E$*)\|\|((E’=E)&&(d’<d))*  *t=0;*  *E=E’;*  *d=d’;*  $F^{*}=F'$*;*  endif  *t=t+1;*  if *t==*$t_{max}$ break;  endfor  return *F**;  End |
| --- |

TABLE 1
Description of imp-LVW

| Algorithm: Imp-LVW  Input:  dataset *X*;  input feature set *F*;  maximum loop number $t_{max};$  Begin  $F^{*}=F$*;*  *d*=21;  Initialize feature weight *w*, for each$f\in F$*,* $w_{f}=1$(where \|*w*\|=\|*F*\|);  generate twenty-one feature subsets by feature weights $w$ using randomize method;  calculate the weight value of each feature *f*$(f\in F)$ using  $w_{f0}=\sum_{m=1}^{21} {(Accu}_{{ft}_{m}}*{flag}_{m})$  where ${flag}_{m}=\left\{ \begin{aligned} 1,if f\in{ft}_{m} \\ 0,else \end{aligned} \right.$  ${(Accu}_{{ft}_{m}}$ is the classification accuracy of subset *m* with SVM model);  $T=\left\vert F \right\vert\times21;$  For *loopnum*=1: *T*  generate feature subset $F^{'}$with 21 features by feature weights $w^{32}$using randomize method;  delete duplicate features from $F^{'}$;  $d^{'}=\left\vert F^{'} \right\vert;$  *E’* is the classification accuracy of $F^{'}$on SVM model;  If *(*$E^{'}<E$*)\|\|((E’=E)&&(d’<d))*  *t=0;*  *E=E’;*  *d=d’;*  $F^{*}=F'$*;*  $w_{f}$*=*$w_{f}+E'$*;*  endif  *t=t+1;*  if *t==*$t_{max}$ break;  endfor  return *F**;  End |
| --- |
